# The effect of electrical neurostimulation on collateral perfusion during acute coronary occlusion

**DOI:** 10.1186/1471-2261-7-18

**Published:** 2007-06-27

**Authors:** Jessica de Vries, Rutger L Anthonio, Mike JL DeJongste, Gillian A Jessurun, Eng-Shiong Tan, Bart JGL de Smet, Ad FM van den Heuvel, Michiel J Staal, Felix Zijlstra

**Affiliations:** 1 Dept of Cardiology, Thoraxcenter, University Medical Centre Groningen, University of Groningen, Groningen, The Netherlands; 2 Dept of Neurosurgery, University Medical Centre Groningen, University of Groningen, Groningen, The Netherlands

## Abstract

**Background:**

Electrical neurostimulation can be used to treat patients with refractory angina, it reduces angina and ischemia. Previous data have suggested that electrical neurostimulation may alleviate myocardial ischaemia through increased collateral perfusion. We investigated the effect of electrical neurostimulation on functional collateral perfusion, assessed by distal coronary pressure measurement during acute coronary occlusion. We sought to study the effect of electrical neurostimulation on collateral perfusion.

**Methods:**

Sixty patients with stable angina and significant coronary artery disease planned for elective percutaneous coronary intervention were split in two groups. In all patients two balloon inflations of 60 seconds were performed, the first for balloon dilatation of the lesion (first episode), the second for stent delivery (second episode). The Pw/Pa ratio (wedge pressure/aortic pressure) was measured during both ischaemic episodes. Group 1 received 5 minutes of active neurostimulation before plus 1 minute during the first episode, group 2 received 5 minutes of active neurostimulation before plus 1 minute during the second episode.

**Results:**

In group 1 the Pw/Pa ratio decreased by 10 ± 22% from 0.20 ± 0.09 to 0.19 ± 0.09 (p = 0.004) when electrical neurostimulation was deactivated. In group 2 the Pw/Pa ratio increased by 9 ± 15% from 0.22 ± 0.09 to 0.24 ± 0.10 (p = 0.001) when electrical neurostimulation was activated.

**Conclusion:**

Electrical neurostimulation induces a significant improvement in the Pw/Pa ratio during acute coronary occlusion.

## Background

Cardiovascular disease is a leading cause of mortality and morbidity. Percutaneous coronary interventions (PCI) and coronary artery bypass grafting (CABG) are established revascularization modalities, yet for some patients PCI and CABG are not an option, or do not result in complete revascularization. The report of the ESC joint study group for refractory angina define refractory angina as a chronic condition which cannot be controlled by medical therapy nor by revascularization, with an estimated prevalence of 30–50 000 patients/year in Europe [1]. One of the treatment options for this patient category is electrical neurostimulation, either transcutaneous electrical nerve stimulation (TENS) with level B evidence, or spinal cord stimulation (SCS) with level A evidence [1]. Patients describe the sensation of electrical stimulation as a tickling, pleasant feeling, in particular during angina. Small randomized trials have demonstrated a safe reduction in angina by electrical neurostimulation, enabling patients to prolong exercise without aggravating myocardial ischaemia. [2-6]

The effect of electrical neurostimulation on coronary flow has been studied in two mechanistic trials. The first trial showed that electrical neurostimulation increased resting coronary flow velocity in a not significantly diseased coronary artery [7]. The second trial used Doppler flow wires in two coronary arteries and showed a decreased coronary flow in the stenotic artery with a concomitant increase in the nonstenotic artery, accompanied by a decrease in ST-T segment depression [8]. This suggests that the myocardial area jeopardized by ischaemia is supplied with blood by an alternative pathway through the coronary collateral circulation [9]. In a pilot study we observed trends that seemed to confirm this hypothesis [10]. In this study the collateral perfusion was determined in an acute coronary occlusion model, during PCI. The collateral perfusion as Pw/Pa ratio can be calculated by dividing the wedge pressure during balloon inflation by the aorta pressure [11]. Pharmacological intervention with adenosine was shown to improve collateral perfusion with ± 10%[12]. In the present study we thought to investigate the effect of electrical neurostimulation on collateral perfusion, defined as a difference in Pw/Pa ratio.

## Methods

### Study population and Design

**Figure 1:**
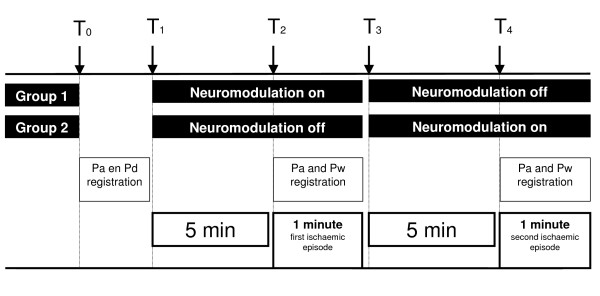
**Study protocol**. At baseline the Pd/Pa ratio over the stenosis was measured. The patients were divided in two groups, the sequence of (in)active electrical neurostimulation was randomly assigned. Electrical neurostimulation was (in)active for 5 minutes, and continued during a 1 minute inflation. Two sequences of 5 + 1 minute were performed, one for dilation with a balloon and one for stent delivery. During the inflations aortic pressure and wedge pressure were recorded. Pd = pressure distal of the stenosis in resting conditions; Pa = aortic pressure; Pw = wedge pressure: pressure distal to the inflated balloon

Between January 2006 and May 2006, 60 consecutive patients with stable angina NYHA class II-III and non-invasive evidence of myocardial ischemia, planned for elective PCI were included. The sample size of this study was calculated with power analysis, based on the data of our pilot study [10]. The coronary artery anatomy had to be suitable for a pressure wire. Patients with a recent myocardial infarction (< 2 weeks), prior CABG, unstable angina, conduction disturbances, pacemaker, or Implantable Cardioverter Defibrillator were excluded. All patients had a normal left ventricular ejection fraction and were without clinical signs of heart failure and without chronic obstructive or restrictive pulmonary disease. Approval of the institutional review board was obtained, the patients were fully informed about the study procedure. Informed consent was obtained from all patients. The patients were randomly divided into two groups, with different sequences of electrical neurostimulation active and off, as shown in Figure 1.

### Percutaneous Coronary Intervention (PCI) procedure

All medication was continued until cardiac catheterization. All patients received aspirin (600 mg) and clopidogrel (600 mg) orally the day before PCI. At the beginning of the catheterization, all patients received heparin (5000 IU) intravenously as a bolus. In all patients cardiac catheterization was performed using the percutaneous femoral approach. A 6F sheath was inserted into the right femoral artery. A 6F guiding catheter was used for the insertion of a 0.36-mm (0.014-in) fiberoptic pressure-monitoring guide wire (Pressureguide, Radi Medical Systems AB, Uppsala, Sweden). The pressure wire was respectively, set at zero, calibrated, advanced through the catheter, equilibrated, introduced into the coronary artery, and positioned distal to the stenosis as previously described by Pijls *et al*[11]. This technique has been shown to have a low variability and an excellent reproducibility [11,13]

### Transcutaneous Electrical NeuroStimulation (TENS)

The patients were treated with conventional TENS providing a continuous flow of symmetrical rectangular biphasic pulses (Schwa Medico, Pierenkemper GMBH; Wetzlarerstr. 41–43, D-35630 Ehringhausen, Germany). The frequency of stimulation was 100 Hz and the pulse-width 200 microsec. Before the PCI procedure the TENS electrodes were applied to the chest; the current was then increased to assess the motor threshold for each patient (i.e. the maximal tolerable stimulation without muscle activation). Active TENS was set at 90% of motor threshold (expressed in mA) [14]. Patients experienced the sensation of electrical neurostimulation in the area, where the electrodes were placed. Electrical neurostimulation produces electrical disturbance on the ECG, although the cardiac rhythm can still be observed reliably, accurate ST shift analysis is not feasible. During TENS off, the stimulator was switched off, with the electrodes still applied to the chest.

### Intra Coronary (IC) Pressure Measurements

At baseline the IC pressure drop over the stenosis (Pd/Pa ratio) was determined by simultaneous measurement of aortic pressure (Pa, mm Hg) and the distal coronary artery pressure (Pd mm Hg), under resting conditions, no hyperaemia was induced. The IC collateral flow (Pw/Pa ratio) was determined by simultaneous measurement of aortic pressure (Pa, mm Hg) and the distal coronary artery pressure during balloon occlusion (Pw, mm Hg) [11]. Pa was measured at the tip of the guiding catheter; Pw was measured with the pressure wire. Central venous pressure was assumed to be 5 mm Hg.

### Coronary angiography analysis

Angiograms were stored on digital media and analyzed visually for information of the number of diseased vessels. Collateral filling was graded according to the Rentrop classification: 0 = no filling of any collaterals, 1 = filling of side branches of the coronary artery by collaterals without visualization of the epicardial segment, 2 = partial filling of the epicardial coronary artery by collaterals, 3 = complete filling of the epicardial coronary artery by collaterals [15].

### Statistical analysis

Nominal variables were compared using the Mann Whitney U test. Continuous, normally distributed variables are expressed as mean ± SD, these variables were compared between group 1 and 2 using the Student t-test. The Pw/Pa ratio during first and second ischaemic episode were compared using student t-test for paired data. Relations between the angiographic presence of collaterals or the number of diseased vessels and the Pw/Pa ratio were investigated, using the highest value of Pw/Pa ratio per patient. Additional analyses were performed, to compare characteristics of patients with and without an effect of electrical neurostimulation. Patients were dichotomized for absolute increase of Pw/Pa ratio of more or less than the mean Pw/Pa ratio value. All tests were two-sided and a p-value of <0.05 was considered significant. Statistical analysis was performed by using SPSS v12.0 software (SPSS Inc. Chicago, IL, USA).

## Results

### Study population

From all 60 patients evaluable measurements were obtained. All patients underwent both ischaemic episodes, and tolerated the full 60 seconds of the two episodes. The mean age of the 60 patients was 67 ± 10 years, 30% of the patients were female and approximately half of the target vessels were left anterior descending arteries (LAD). There were no differences in baseline characteristics between the two groups (Table 1). The settings of electrical neurostimulation were similar in the two groups, with a motor threshold in group 1 of 7.8 ± 2.7 mA, and in group 2 of 7.3 ± 2.1 mA (p = 0.57).

**Table 1:** Baseline characteristics

	Group 1	Group 2	P-value
	N = 30	N = 30	
Age	68 ± 9	66 ± 11	0.46
BMI	28 ± 4	28 ± 4	0.77
Female	9 (30)	9 (30)	1.00
Culprit vessel			0.86
LAD	14 (47)	13 (43)	
CX	9 (30)	11 (37)	
RCA	7 (23)	6 (20)	
Number of diseased vessels			0.55
1 vessel disease	12 (40)	14 (47)	
2 vessel disease	12 (40)	13 (43)	
3 vessel disease	6 (20)	3 (10)	
Rentrop class			0.49
0	25 (83)	27 (90)	
1	1 (3)	2 (7)	
2	3 (10)	1 (3)	
3	1 (3)	0	
β-blockers	22 (73)	27 (90)	0.10
Calcium-blockers	14 (47)	11 (37)	0.44
Long acting nitrates	9 (30)	6 (20)	0.38
Ace inhibitors	10 (33)	9 (30)	0.78
Diuretics	6 (20)	10 (33)	0.25
Aspirin	23 (77)	24 (80)	0.76
Statins	22 (73)	20 (66)	0.58
Hypertension	13 (43)	13 (43)	1.00
Hypercholesterolaemia	6 (20)	8 (27)	0.55
Diabetes	8 (27)	6 (20)	0.55
Smoking	4 (13)	7 (23)	0.32
Family history	4 (13)	8 (27)	0.20

Continuous variables are presented as mean ± SD, categorical variables are presented as number (%). BMI: body mass index; LAD: Left anterior descending artery; CX: circumflex artery; RCA: right coronary artery

### IC Pressure measurements

**Figure 2:**
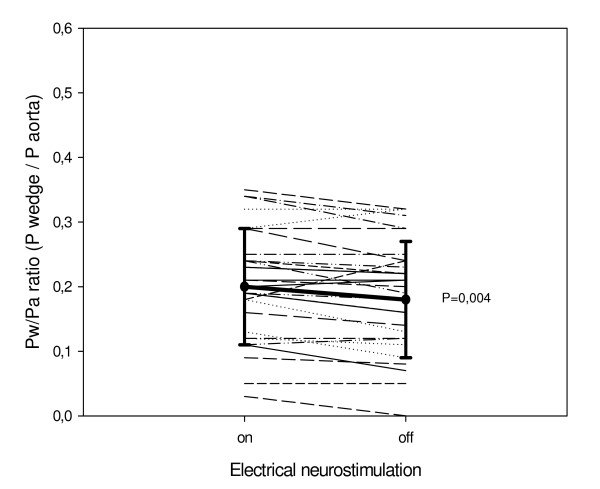
**Individual Pw/Pa ratio values and mean ± SD of group 1**. The individual values of collateral flow index are presented with the mean value and the standard deviation. Pw/Pa ratio = pressure distal to inflated balloon/aortic pressure.

**Figure 3:**
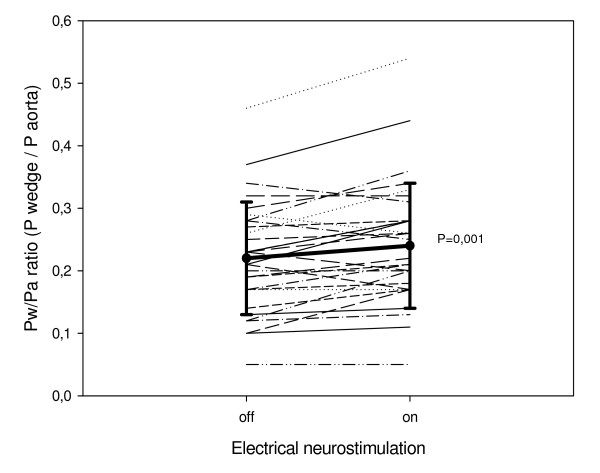
**Individual Pw/Pa ratio values and mean ± SD of group 2**. The individual values of collateral flow index are presented with the mean value and the standard deviation. Pw/Pa ratio = pressure distal to inflated balloon/aortic pressure.

The Pd/Pa ratio was similar in the two groups (0.77 ± 0.22 and 0.74 ± 0.23, p = 0.59). With active TENS, the Pw/Pa ratio of all 60 patients together increased significantly with 13 ± 21% (from 0.21 ± 0.09 to 0.22 ± 0.09, p < 0.001). Figure 2 and 3 show the Pw/Pa ratio values of all patients during the two ischaemic episodes. Figure 2 shows a significant decrease of the Pw/Pa ratio in group 1 from 0.20 ± 0.08 to 0.19 ± 0.09, when electrical neurostimulation is switched off (p = 0.004). Figure 3 shows a significant increase in Pw/Pa ratio in group 2 from 0.22 ± 0.09 to 0.24 ± 0.10, when electrical neurostimulation is activated (p = 0.001). Separate analyses of the change in Pa and Pw in both groups showed Pa did not change significantly in group 1 (99 ± 18 mm Hg to 98 ± 16 mm Hg, p = 0.53), nor in group 2 (101 ± 11 mm Hg to 99 ± 13 mm Hg, p = 0.28). In group 1 Pw changed significantly (20 ± 9 mm Hg to 19 ± 9 mm Hg, p = 0.02), and there was a trend in group 2 (22 ± 9 mm Hg to 25 ± 11 mm Hg, p = 0.07).

### Coronary Angiographic analysis

**Figure 4:**
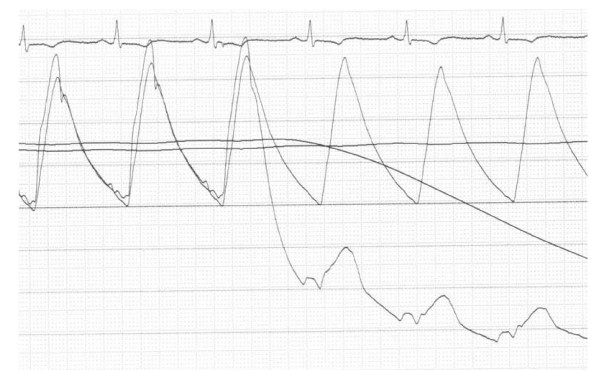
**Pressure recordings of a patient**. The pressure recordings are shown, when the pressure wire passed the stenosis. The line going down represents the distal (wedge) pressure, the other line represents the aortic pressure. On top of the figure one lead of the electrocardiogram is shown.

**Figure 5:**
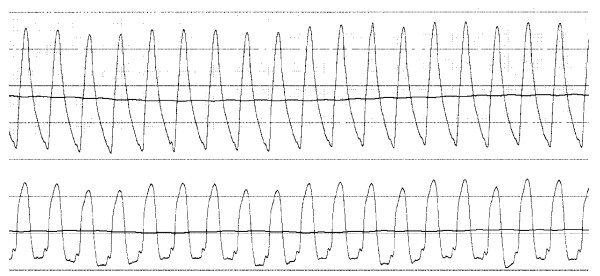
**Pressure recordings of a patient**. Showing the aortic pressure and distal coronary wedge pressure during the first ischaemic episode. The lower line represents the wedge pressure (Pw), characterized by a shape that shows that the diastolic pressure is most compromised. The upper line represents the aortic pressure (Pa). This Pw/Pa ratio is 0.22.

Figure 4 shows the recordings of the aortic pressure and distal coronary pressure, when the wire passed the stenosis, the pressure drop over the stenosis under resting conditions was 0.28. Figure 5 shows the recording of the Pw/Pa ratio during the 60 seconds of balloon occlusion, the collateral flow index was 0.22.

As presented in table 1 and 2, there were 52 patients without signs of collaterals on the coronary angiogram (Rentrop class 0), there were 3 patients with Rentrop class 1, 4 patients with Rentrop class 2 and 1 patient with Rentrop class 3. Patients with angiographic signs of collaterals have a significantly higher Pw/Pa ratio (0.22 ± 0.01 vs 0.31 ± 0.04, p = 0.02). The study group consisted of 26 patients with single vessel disease, 25 patients with two vessel disease and 9 patients with three vessel disease. Patients with one and two vessel disease have comparable Pw/Pa ratio values (0.23 ± 0. 02 vs. 0.24 ± 0.01) and patients with three vessel disease have lower Pw/Pa ratio values (0.19 ± 0.02), albeit without a significant difference between the three categories (p = 0.32).

**Table 2:** Characteristics of groups dichotomized for effect of electrical neurostimulation

	Effective	Ineffective	P-value
	N = 28	N = 32	
Age	66 ± 10	67 ± 11	0.81
BMI	29 ± 5	27 ± 3	0.17
Female	5 (18)	13 (41)	0.06
Culprit vessel			0.84
LAD	14 (50)	13 (41)	
CX	5 (18)	8 (25)	
RCA	9 (32)	11 (34)	
Number of diseased vessels			0.01
1 vessel disease	10 (36)	16 (50)	
2 vessel disease	17 (61)	8 (25)	
3 vessel disease	1 (4)	8 (25)	
Rentrop class			0.28
0	25 (89)	27 (84)	
1	0	3 (9)	
2	2 (7)	2 (6)	
3	1 (4)	0	
B-blocker	26 (93)	23 (72)	0.04
Ca-blocker	9 (32)	16 (50)	0.17
Long acting nitrates	9 (32)	6 (19)	0.24
Ace inhibitor	9 (32)	10 (31)	0.94
Diuretics	9 (32)	7 (22)	0.37
Aspirin	21 (75)	26 (81)	0.56
Statins	20 (71)	22 (69)	0.82
Hypertension	14 (50)	12 (38)	0.33
Hypercholesterolaemia	9 (32)	5 (16)	0.14
Diabetes	5 (18)	5 (16)	0.35
Smoking	7 (25)	4 (13)	0.22
Family history	6 (21)	6 (19)	0.80

Continuous variables are presented as mean ± SD, categorical variables are presented as number (%). BMI: body mass index; LAD: Left anterior descending artery; CX: circumflex artery; RCA: right coronary artery. Twenty eight patients with Pw/Pa ratio changes in excess of the mean group value were compared with 32 patients with Pw/Pa ratio changes less than the mean value.

### Subgroup analysis

Electrical neurostimulation resulted in 41 out of the 60 patients (68%) in improvement of the Pw/Pa ratio. The mean absolute change in Pw/Pa ratio in the 60 patients was 0.02 ± 0.03. There were 28 patients with a Pw/Pa ratio change in excess of this mean value, they were compared with 32 patients with a Pw/Pa ratio change less than the mean value. Additional analysis of these groups showed similar values for the stenosis related pressure drop under resting conditions (0.72 ± 0.26 vs. 0.79 ± 0.18, p = 0.24), and for electrical neurostimulation characteristics as reflected by motor threshold (7.4 ± 2.3 mA vs. 7.7 ± 2.5 mA, p = 0.66). Baseline clinical and angiographic characteristics of patients with and patients without an effect of electrical neurostimulation are shown in table 2.

## Discussion

In this study of 60 patients with significant coronary artery disease, the Pw/Pa ratio increased when electrical neurostimulation was active for 5 + 1 minutes and decreased when electrical neurostimulation was off for 5 + 1 minutes. The functional collateral perfusion was assessed by distal coronary pressure measurements during acute coronary occlusion. The target lesions of the patients included in this study were all visually significant stenoses on the coronary angiogram, also shown by non-invasive evidence of myocardial ischemia and reflected by the Pd/Pa ratio over the stenoses under resting conditions.

### Electrical neurostimulation and perfusion

Some studies found no effect of electrical neurostimulation on the coronary or systemic haemodynamics and postulated an anti-ischaemic effect secondary to a decrease in myocardial oxygen consumption [16-18]. Where other studies have shown that electrical neurostimulation reduced afterload, following systemic vasodilatation [19] and a fall in systolic blood pressure possibly by reduced sympathetic activity [20]. Our results show no significant change of the systolic blood pressure (Pa) by electrical neurostimulation in this study protocol. Other findings have resulted in a hypothesis that electrical neurostimulation has anti-ischaemic effects by increasing collateral perfusion [8]. Other mechanisms that may explain the anti-ischaemic effects of electrical neurostimulation as reflected by less ST-T segment depression, include homogenization of myocardial perfusion documented in a study using positron emission tomography,[21] and an increase in intracoronary blood flow velocity measured with a Doppler technique in patent coronary arteries [7,8]. In our pilot study we found a trend towards an increase of the Pw/Pa ratio by electrical neurostimulation, [10] and therefore we performed this study, to prove or disprove the concept that electrical neurostimulation increases functional collateral perfusion as represented by the Pw/Pa ratio.

### Collateral perfusion

Collateral perfusion offers an alternative pathway of blood supply towards the ischaemic myocardial area, thereby decreasing myocardial ischaemia. The often limited functional significance of collateral blood supply makes improvement of coronary collateral circulation an interesting treatment target. Many different diagnostic methods have been developed to investigate the functional significance of coronary collaterals. A classification system for collateral filling from patent vessels to diseased vessels was developed by Rentrop using coronary angiography [15]. This technique has a major limitation, as only spontaneous visible collateral vessels exceeding 100 microns in diameter can be visualized [22]. A rather crude measure for coronary collaterals is the absence of myocardial ischaemia during coronary occlusion [23,24]. More accurate, non-invasive diagnostic techniques to investigate coronary collaterals are positron emission tomography, [25,26] and myocardial contrast echocardiography [27]. Hemodynamic variables, reflecting the collateral circulation are excellent markers of the functional significance of collaterals, provided that these variables are measured in a reproducible and accurate manner [13,28,29]. Several studies have compared the various methods and have investigated whether different methods were complementary for the assessment of collaterals [30-33]. In a trial to measure the effect of adenosine on the collateral perfusion, pressure as well as flow velocity recordings were assessed [12]. Both outcome measures showed similar changes in collateral perfusion. Pharmacological intervention with adenosine was shown to have a comparable impact on collateral perfusion as we have found with electrical neurostimulation.

### Mechanism of collateral perfusion improvement

De Marchi et al found that sympathetic stimulation using the cold pressor test increased coronary collateral flow [34]. Pohl et al found that exercise increased coronary collateral perfusion, [35] and that beta-blockade tended to reduce these effects, in agreement with previous clinical studies [34,36]. The few experimental investigations on vasomotor properties of coronary collateral vessels suggest that the sympathetic nervous system might predominantly exert a β-receptor-mediated (i.e. vasodilative) effects on coronary collaterals [37,38].

Electrical neurostimulation was found to decrease total body norepinephrine (NE) by 18% (p = 0.02) [39]. During pacing NE levels increased by 47% (p = 0.02), while total cardiac norepinephrine spillover remained unchanged during pacing and active SCS. They suggest that the antiischemic effects of electrical neurostimulation are not exerted via cardiac sympathetic activity, although the overall sympathetic activity is decreased. Recently, 5 minutes of electrical neurostimulation at 90% of motor threshold was found to induce cutaneous vasodilation, mediated via antidromic activation of sensory fibers [40]. Whether this mechanism accounts for the increase in collateral perfusion has not been elucidated yet.

### Limitations

In clinical practice electrical neurostimulation is often used for 1–3 hours. Since neurostimulation was active in our trial for only 5 + 1 minutes, only short-term effects of electrical neurostimulation can be studied. That PCI can be used as an investigational tool of acute myocardial ischemia was already proven in 1986 by Serruys [41]. Since electrical neurostimulation disturbes the ECG, we were not able to perform additional ST shift analyses. Nevertheless, the relation between collateral perfusion and ischemia has already been investigated by others [13,32,42].

We assumed the central venous pressure (CVP) to be 5 mm Hg, but we did not measure the actual CVP in the individual patients. Nevertheless, this seems a reasonable assumption as we excluded all patients with clinical signs of heart failure, obstructive or restrictive pulmonary disease, and all patients had normal left ventricular function.

### Clinical implications and future research

Our results indicate an acute positive effect of electrical neurostimulation on collateral perfusion. Our data do not allow conclusions with regard to the magnitude and/or durability of the collateral flow augmentation induced by electrical neurostimulation, but our data should be interpreted as "proof of concept", that electrical neurostimulation alleviates myocardial ischemia by an increase in collateral perfusion. Further studies are needed to investigate the magnitude and durability of the effects of neurostimulation on collateral perfusion.

## Competing interests

The author(s) declare that they have no competing interests.

## Pre-publication history

The pre-publication history for this paper can be accessed here:


